# Sequential pattern transformer (SPT): a generative and interpretable framework for predicting disease trajectories

**DOI:** 10.1007/s00521-025-11695-4

**Published:** 2026-02-03

**Authors:** Mohammad Assadi Shalmani, Masoud Khani, Amirsajjad Taleban, Zihao Yi, Jennifer T. Fink, Christopher E. Weber, Qiang Lu, Jake Luo

**Affiliations:** 1 Health Informatics Program, Zilber School of Public Health, University of Wisconsin-Milwaukee, Milwaukee, WI, USA; 2 Department of Health Informatics and Administration, College of Health Sciences, University of Wisconsin-Milwaukee, Milwaukee, WI, USA; 3 Department of Pediatrics and Internal Medicine, Ascension Columbia St. Mary’s Hospital, Milwaukee, WI, USA; 4 Department of Computer Science and Technology, China University of Petroleum, Beijing, China

**Keywords:** Disease trajectory prediction, Transformer architecture, Generative AI, Healthcare informatics, Explainable AI (XAI), Diabetes mellitus, Clinical decision support, Sequential pattern mining

## Abstract

The effective integration of artificial intelligence into clinical workflows requires models that go beyond simple prediction to generate comprehensive, explainable, and actionable disease trajectories. Addressing the limitations of opaque deep learning architectures and the noise inherent in electronic health records, we introduce the sequential pattern transformer (SPT), a novel framework that synergizes sequential pattern mining with generative transformer modeling. Using four years of inpatient data from 258,460 type 2 diabetes patients, we applied the PrefixSpan algorithm to distill noisy diagnostic histories into a curated vocabulary of 95,630 statistically validated disease progression patterns. A decoder-only transformer was trained exclusively on these evidence-based sequences to learn the temporal dynamics of disease evolution. This pattern-guided approach shifts the modeling paradigm from classification to probabilistic trajectory generation. The model achieved a robust 85.78% Top-5 accuracy, significantly outperforming a standard LSTM baseline (71.47%). Beyond predictive accuracy, the framework constructs a dynamic Disease Atlas, a branching tree structure that visualizes likely future pathways, augmented by multi-level explainable AI (XAI) including learned clinical clusters, SHAP-based feature attribution, and counterfactual simulations. Crucially, this methodology is domain-agnostic and capable of efficient fine-tuning, making it a transferable solution for adapting to diverse clinical conditions and local hospital settings. SPT thus offers a transparent, robust, and scalable framework for mapping the complex temporal dynamics of disease, bridging the gap between high-performance AI and interpretable clinical application.

## 1 Introduction

Chronic non-communicable diseases are the leading cause of mortality worldwide, accounting for approximately 70% of all deaths globally [1]. The health and economic burden of these conditions is substantial, with projected global costs reaching $47 trillion by 2030 [2]. For instance, diabetes mellitus affects 537 million adults [3], directly causing 1.6 million deaths annually [4]. In the United States, diabetes incurs annual costs exceeding $327 billion [5] reflecting a staggering economic impact primarily driven by direct medical expenses [6].

Despite this burden, current care models for these chronic conditions remain predominantly reactive, typically intervening only after complications arise [7]. This paradigm misses critical opportunities to prevent or slow disease progression, leading to unnecessary patient suffering and escalating healthcare costs [8]. A fundamental shift to a proactive model is urgently needed, hinging on our ability to accurately forecast individual disease trajectories to guide timely, preventive interventions.

Predictive modeling holds significant promise for enabling this shift, but existing methods face critical limitations. Conventional machine learning algorithms (e.g., logistic regression, random forests) are designed for static, tabular data and fail to capture the dynamic, temporal nature of disease progression [9–11]. While deep learning models like Recurrent Neural Networks (RNNs) and Long Short-Term Memory Networks (LSTMs) were introduced to handle sequences, their susceptibility to the vanishing gradient problem limits their ability to learn long-range dependencies in extensive patient histories [12–14], a crucial requirement for modeling chronic disease.

Transformer architectures have recently emerged as a more powerful alternative, using self-attention mechanisms to effectively capture complex, long-range relationships in longitudinal patient records [15–17]. However, when applied to raw clinical data, standard transformers are not without challenges. They are vulnerable to learning spurious correlations from noisy, high-dimensional event sequences and can perpetuate systemic biases present in healthcare data [18, 19]. Furthermore, the difficulty in understanding how these models work internally remains a significant barrier to clinical trust and implementation, as translating their complex mechanisms into actionable insights is a persistent challenge [20].

To address these dual challenges of data noise and a lack of interpretability, we introduce the Sequential Pattern Transformer (SPT), a novel framework that synergistically integrates sequential pattern mining with a generative transformer. Our approach first uses sequential pattern mining to distill noisy patient histories into a vocabulary of statistically validated, frequent disease pathways. This process acts as a powerful noise-reduction and feature engineering step, providing a high-signal foundation for the transformer to mitigate the risk of learning spurious correlations. By employing pattern mining as an intelligent preprocessing step, our method is designed to enhance predictive accuracy while embedding multi-level interpretability, from population-level disease relationships to individual prediction attributions.

## 2 Related work

The pursuit of proactive disease management has driven significant advancements in predictive modeling for sequential health data. However, progress has been marked by a persistent tension between architectural power and the inherent challenges of real-world clinical data. This section reviews key advancements not as separate research areas, but as an evolving narrative that reveals the critical, unaddressed gap our work aims to fill.

### 2.1 The evolution from recurrent models to transformers

Early deep learning research turned to architectures designed for sequential data. Lipton et al. [21] pioneered the use of LSTM networks on clinical time series, demonstrating improved capture of temporal dependencies compared to static models. This was followed by influential, recurrent-based models like RETAIN, which enhanced performance and explainability with attention mechanisms [22], and Doctor AI, which modeled disease progression with an RNN-based approach [12]. Others like Dipole [23] and KAME [24] further refined attention-based RNNs on diabetes datasets. Although direct comparison is challenging due to varied evaluation metrics, dataset, and tasks, these foundational RNN-based approaches established important benchmarks and demonstrated the potential of sequential modeling for healthcare. However, they consistently struggled to learn the very long-range dependencies present in patient histories spanning years and numerous clinical encounters.

### 2.2 Transformer-based approaches in healthcare

The advent of transformer architectures marked a significant advancement in sequence modeling [25], prompting their adoption in healthcare. Shang et al. [26] were among the first to apply transformers to EHRs, outperforming recurrent models in medication recommendation tasks with an F1-score of 0.62. Their work showcased the power of self-attention mechanisms in capturing complex clinical data relationships. Li et al. extended this with BEHRT [17], a BERT-based model treating diseases as tokens in a sequence, achieving an average precision score of 0.462 for predicting next diagnoses across multiple conditions. However, BEHRT focused primarily on classification, limiting its ability to generate alternative progression pathways. Med-BERT [15] further demonstrated the power of transformers for clinical classification tasks, employing encoder-only architecture for effective clinical representation learning. These transformer-based healthcare models established the foundation for applying attention mechanisms to clinical data and achieved strong performance on various prediction tasks.

More recent approaches have continued this trend. For example, Tran et al. [27] presented an explainable stacking ensemble integrating a Transformer with other machine learning models to predict diabetes complications. While this method achieved a high AUC score of 0.86, it remained confined to classification. This highlights a persistent limitation: while classification models can predict the *risk* of a future event, they cannot generate the *sequence* of probable events. This limits their clinical utility, as proactive care requires understanding the likely pathways toward a complication, not just knowing it might occur.

### 2.3 Gaps in current approaches and research opportunity

The reviewed literature highlights a clear opportunity to enhance the application of Transformers in healthcare. A promising, less-explored direction is to move beyond single-event classification and leverage the generative capabilities of these models to forecast entire patient trajectories. Furthermore, grounding their powerful learning mechanisms in high-signal, evidence-based clinical pathways, rather than raw event sequences, can improve both robustness and transparency. Our work is situated in this research gap, aiming to develop a framework that is not only predictive but also generative and inherently interpretable, aligning state-of-the-art deep learning with the practical needs of clinical decision support.

## 3 Methodology

**Fig. 1:**
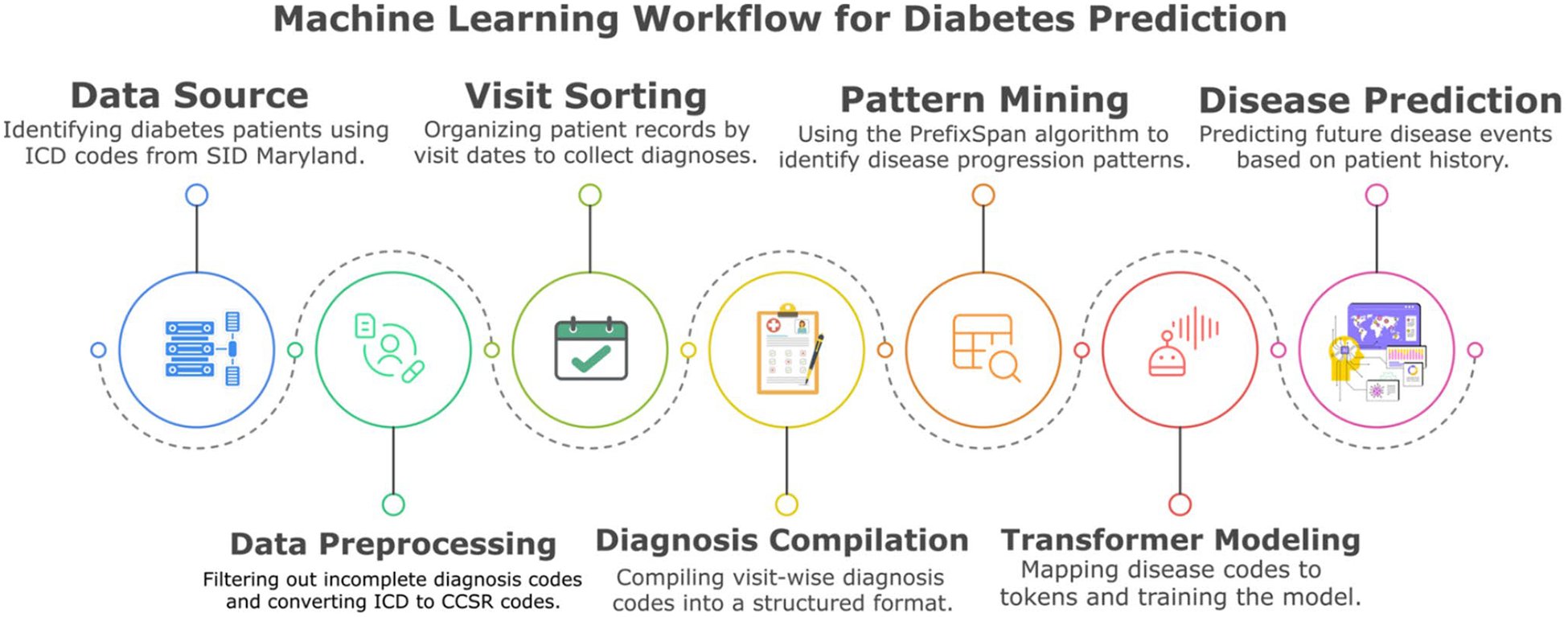
SPT Methodology Pipeline. SPT pipeline consisting of seven sequential stages: Data Source Identification (identifying diabetes patients from Maryland SID database), Data Preprocessing (filtering incomplete diagnoses and converting International Classification of Diseases (ICD) to Clinical Classifications Software Refined (CCSR) codes), Visit Sorting (organizing patient records by visit dates), Diagnosis Compilation (compiling visit-wise diagnosis codes into structured format), Pattern Mining (using PrefixSpan algorithm to identify disease progression patterns), Transformer Modeling (mapping disease codes to tokens for model training), and Disease Prediction (predicting future disease events based on patient history)

Our methodology follows a structured pipeline comprising seven key stages: patient data extraction from the State Inpatient Database (SID) [28], preprocessing of clinical records, sorting visits based on their time, pattern mining to identify frequent disease trajectories, data transformation into sequential text-based representations, training of a decoder-only transformer model, and disease progression prediction along with implementation of Explainable AI(XAI) techniques to ensure interpretability and clinical utility. Figure 1 shows the complete methodological pipeline.

### 3.1 Data source

This study utilized the SID from the Healthcare Cost and Utilization Project (HCUP) [29] for Maryland, covering the years 2016 to 2019. The SID provides comprehensive information on inpatient hospital stays, including patient diagnoses, procedures, admission/discharge dates, and demographic features. It captures data from all inpatient stays in participating states, making it a representative sample of hospitalizations.

To focus on type 2 diabetes mellitus patients, we filtered records containing the International Classification of Diseases (ICD)-10 code E11.X, resulting in 258,460 unique patient records. Invalid patient identifiers were removed to ensure accurate tracking of individual trajectories. The four-year period enabled observation of long-term disease progression and recurring hospitalizations.

The baseline demographic of this cohort is detailed in Table 1. The average age of the cohort was 66.2 years, with a balanced gender distribution (50.4% female). The population was racially diverse, composed primarily of White (52.9%) and Black (37.5%) patients, with Medicare (60.2%) being the most common primary insurer.

### 3.2 Data preprocessing and sequence construction

The initial dataset was preprocessed to ensure data quality and create a format suitable for sequence modeling. First, to reduce noise, records containing unknown or unspecified diagnoses were excluded. Next, to address the high dimensionality of the raw data while preserving clinical meaning, we transformed the granular ICD-10 diagnosis codes into 225 broader categories using the Clinical Classifications Software Refined (CCSR) [30]. This aggregation is crucial as it makes the dataset more tractable for modeling and enhances the interpretability of the results by grouping related conditions into cohesive clinical concepts.

**Fig. 2:**
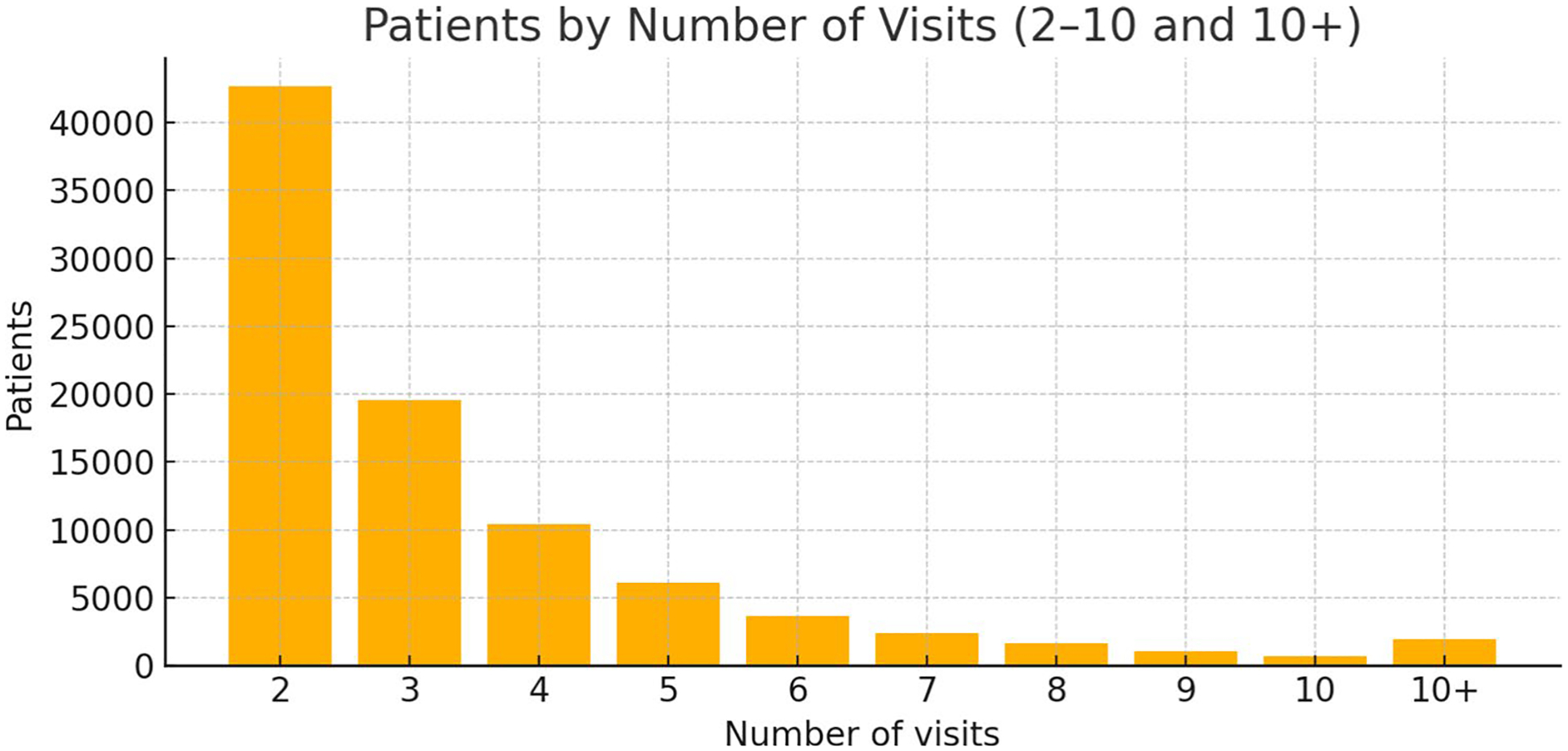
Patient visit counts shown as individual bars for 2–10 and a single ‘10 + ‘ bar representing ≥ 11 visits. Summary: N = 90,078; total visits = 313,145; median 3; mean 3.48 (SD 2.61). Patients with 2–5 visits comprise 87.3% of the cohort, while those with ≥ 10 visits represent 2.94% of patients and contribute 11.4% of encounters

Following the cleaning and standardization of diagnosis codes, the preprocessed records for each patient were structured into a continuous, sequential format for modeling. First, all hospital records for a unique patient were collected and sorted chronologically by their visit date. From this ordered timeline of encounters, we constructed a single disease trajectory by extracting all CCSR diagnosis codes in the order they appeared across all visits and concatenating them. This process transformed each patient’s history into a single, temporally ordered sequence of disease codes, formally represented as S_p_ = (d_1_,d_2_*,…*, ${\text{d}}_{{\text{k}}_{\text{P}}}$), where K_p_ is the total number of diagnoses recorded for that patient over their entire history. This flat sequence representation treats each diagnosis as a distinct event or “word” in the clinical narrative of the patient, creating a format that is directly suitable for our transformer model to learn the temporal patterns of disease progression. Across all patients, visit counts are concentrated in routine care ranges: 87.3% of patients have 2–5 visits (median 3; mean 3.48, standard deviation 2.61). The cohort also includes extended care histories. 2.94% of patients have ≥ 10 visits and account for 11.4% of all encounters, providing depth for modeling both common and prolonged trajectories. Figure 2 shows the distribution of the patient visits.

### 3.3 Pattern mining

To identify recurring disease sequences, we employed the PrefixSpan algorithm for sequential pattern mining due to its computational efficiency with large datasets and its pattern-projected growth approach [31]. Unlike breadth-first methods such as GSP [32] and SPADE [33] that generate numerous candidate sequences, PrefixSpan focuses on dense pattern regions, making it particularly effective for discovering frequently co-occurring disease trajectories [34].

To prepare the patient data for sequential pattern mining, our first step was to structure it in a way that reflects the natural grouping of diagnoses within distinct clinical encounters. For each patient, we organized their medical history into a chronological sequence of their hospital visits. All diagnosis codes recorded during a single visit were consolidated into a single set or item set. This process transformed each patient’s entire history into an ordered sequence of these item sets, creating a list-of-lists format. Preserving the temporal order of hospital visits. We applied a minimum support threshold of 1000 to focus on clinically significant patterns while filtering out rare sequences. This process yielded 95,630 unique frequent disease sequences. The identified patterns were ranked by frequency to prioritize the most prevalent disease trajectories with substantial clinical relevance. By implementing PrefixSpan, we successfully extracted meaningful sequential patterns that reveal important relationships between diseases across the patient population, providing actionable insights for clinical decision support.

This pattern extraction step is the foundational component of our “pattern-guided” framework and serves a critical dual purpose. First, by enforcing a minimum support threshold, we strategically reduce noise by filtering out rare, idiosyncratic patient trajectories and isolating only the 95,630 statistically validated, common pathways. Second, this process generalizes the core characteristics of disease progression; the model learns from these shared, archetypal sequences rather than overfitting to unique patient histories. This “clean” set of 95,630 patterns becomes the direct input dataset for training our transformer, ensuring it learns from the most prevalent and clinically relevant progressions, as detailed in the following section.

### 3.4 Transformer model

We developed the SPT to address the fundamental challenge of modeling complex temporal dependencies in disease progression trajectories. The model’s core hypothesis is that a patient’s clinical journey exhibits both local sequential patterns and long-range dependencies, which can be effectively captured by a powerful sequence-to-sequence architecture. To this end, the SPT employs a decoder-only transformer architecture, a design inspired by its proven success in generative language modeling and optimized for the unique characteristics of clinical data [25, 35].

#### 3.4.1 Input representation

The model’s operation begins with the input representation. Disease trajectories (i.e., the 95,630 pattern sequences) are encoded through a multi-layered representation scheme. Each of the 225 CCSR disease categories is first mapped to a unique identifier, preserving the discrete nature of diagnostic codes while enabling efficient computation. These identifiers are subsequently transformed into dense d-dimensional embeddings through a learnable lookup table, where d represents the model’s hidden dimensionality. To capture temporal ordering, critical for disease progression modeling, we augment these embeddings with sinusoidal positional encodings that provide position-specific information without introducing learnable parameters that might overfit to specific sequence lengths.

We implement a dynamic approach for sequence-to-sequence learning. Given a patient trajectory of length n, we generate n – 1 training examples by treating each prefix of length k (where 1 ≤ k < n) as input and the subsequent diagnosis as the target. This strategy maximizes data utilization while ensuring the model learns to make predictions from varying historical contexts, a critical capability for clinical deployment where prediction requests may occur at any point in a patient’s journey.

#### 3.4.2 Decoder layer

The core of the SPT is a stack of identical decoder layers, following the canonical transformer design [25]. Each layer is composed of two primary sub-components:

Masked Multi-Head Self-Attention: This mechanism allows the model to dynamically weigh the influence of all previous diagnoses when predicting the next one. The “masking” is critical; it prevents the model from attending to future tokens in the sequence, thereby enforcing the autoregressive property required for step-by-step prediction. The “multi-head” aspect enables the model to capture different types of clinical relationships (e.g., causal, correlational) simultaneously from different representational subspaces.

Position-wise Feed-Forward Network (FFN): This is a fully connected network applied independently to each position’s representation after the attention step. It provides non-linearity and increases the model’s capacity to learn more complex transformations. Both sub-components within each layer are wrapped with residual connections and followed by layer normalization, which are essential for stabilizing the training of deep networks by mitigating the vanishing gradient problem [36]. Finally, after the input sequence is processed through the entire stack of decoder layers, the resulting output representation for the final token is passed to a linear layer and a softmax function. This projects the high-dimensional vector back into the vocabulary space, producing a probability distribution over all possible future disease codes.

This decoder-only design was a deliberate choice stemming from three key considerations: (1) its autoregressive nature is a natural fit for predicting future clinical events based on past observations; (2) it inherently handles the variable-length patient trajectories found in real-world data without requiring padding to a fixed length; and (3) its self-attention weights provide a path to interpretable predictions, allowing clinicians to inspect which past conditions the model deemed most important, a vital feature for building trust and facilitating clinical implementation.

#### 3.4.3 Training methodology

To determine the optimal architecture for our model, we conducted a comprehensive hyperparameter search using Bayesian optimization, implemented with the Optuna framework [37]. We adopted a tiered strategy, defining three distinct studies to explore “small,” “medium,” and “large” model configurations. This approach allowed us to systematically evaluate performance across different scales of model capacity.

Each of the three studies consisted of 50 trials. In each trial, key architectural and training parameters were sampled from the predefined ranges detailed in Table 2. The search space included intelligent structural constraints; for example, the number of attention heads (nheads) was required to be a valid divisor of the model’s embedding dimension (dmodel). The optimization objective was to maximize the Top-1 accuracy on a held-out validation set.

During each trial, models were trained using the Adam optimizer with a cross-entropy loss function. We employed an early stopping mechanism with a patience of 20 epochs to prevent overfitting. This value was chosen to provide a robust balance. It is generous enough to allow models to converge even when validation loss fluctuates, yet restrictive enough to manage computational resources effectively across the 150 total trials.

Following each 50-trial study, the configuration with the best validation accuracy was retrained from scratch on the combined training and validation sets. This final model was then evaluated on the held-out test set to report performance across a suite of metrics.

The trained SPT model outputs a probability distribution over the 225 disease categories for each prediction step. During inference, the model processes patient trajectories of arbitrary length and produces calibrated probability estimates for potential next diagnoses. This probabilistic output enables both point predictions (via argmax) and uncertainty quantification through the entropy of the predicted distribution, providing clinicians with confidence estimates alongside predictions. Training was conducted in parallel on three V100 NVIDIA GPUs.

### 3.5 Model evaluation

To rigorously assess our framework, we first partitioned our dataset into a training set (66,941 sequence), a validation set (9563 sequence), and a test set (19,126 patients). We designed a multi-faceted evaluation process centered on two primary goals: next disease prediction and multi-step disease trajectory generation. The next disease prediction evaluation was itself conducted in two parts. First, we performed an overall performance comparison, where model performance was averaged across all prediction time steps in the test set, to benchmark our SPT model against established sequence modeling baselines: a RNN, a Gated Recurrent Unit (GRU), and a LSTM network. Second, to understand how performance is influenced by the amount of available patient history, we conducted a dedicated analysis of the impact of sequence length, where the model’s predictive accuracy was measured at different positions within a trajectory. For both predictive analyses, we employed a comprehensive suite of metrics. To reflect clinical utility, where a physician is interested in a small set of likely diagnoses, we measured Top-k Accuracy (k = 1, 3, 5). We used Mean Reciprocal Rank (MRR) to evaluate how highly the model ranked the correct diagnosis and Perplexity (PPL) to gauge model confidence. To account for the significant class imbalance in clinical data, we also employed Cohen’s Kappa (Kappa) and the Matthews Correlation Coefficient (MCC), which provide a more robust measure of performance than accuracy alone.

After establishing the superior predictive accuracy of the SPT model in the first task, we proceeded to the second stage: evaluating its capability for multi-step disease trajectory generation. In this analysis, we assessed the standalone generative performance of our best model. We again used PPL to track how the model’s confidence evolved as it generated longer sequences. To measure the factual accuracy and content fidelity of the generated pathways, we calculated Sequence Overlap, which quantifies the proportion of correctly predicted diseases compared to the patient’s true trajectory. Finally, to evaluate the structural coherence of the generated sequences, we adopted metrics from the field of natural language generation, which aligns with our conceptualization of disease progression as a language. Bilingual evaluation understudy (BLEU)-1 measures the precision of individual predicted diseases (or “unigrams”), while Recall-Oriented Understudy for Gisting Evaluation (ROUGE)-L assesses the global integrity of the generated pathway by measuring the longest common subsequence against the true sequence. Together, these metrics provide a holistic view of the model’s ability to generate clinically plausible and structurally sound disease trajectories.

To test the generalizability of our framework, we performed an external validation using the MIMIC-IV [38] dataset. We applied the identical preprocessing pipeline to this new dataset to ensure consistency. Following this, we fine-tuned our best-performing model from the initial experiments on the preprocessed MIMIC-IV data and subsequently reported its performance results.

## 4 Results

### 4.1 Next disease prediction task

#### 4.1.1 Overall performance comparison

In the overall performance comparison for the next disease prediction task, our SPT model demonstrated the most robust performance across all metrics when compared against RNN, GRU, and LSTM baselines, as summarized in Table 3. The best-performing model was SPT-Large, which uses a standard Transformer architecture. It was configured with a model dimension (embedding) of 768, 6 Transformer layers, 16 attention heads, and a feed-forward dimension of 8192.

The best-performing configuration, SPT-Large, achieved a Top-1 accuracy of 41.23%, compared to 31.65% for the strongest baseline model, LSTM. This performance advantage was more pronounced in broader accuracy assessments. For instance, the Top-5 accuracy for SPT-Large was 85.78%, a substantial increase over the 71.47% achieved by the LSTM. These results suggest a superior capacity of the SPT model to place the correct diagnosis within the top-ranked predictions. This trend of superior performance was consistent across the remaining metrics. SPT-Large obtained the highest Mean Reciprocal Rank (MRR) of 0.5897 and the lowest (most favorable) Perplexity (PPL) of 12.34, signifying more accurate ranking and higher model confidence compared to the LSTM’s MRR of 0.4850 and PPL of 28.71. Furthermore, its leading scores on metrics robust to class imbalance, Cohen’s Kappa (0.3027) and the Matthews Correlation Coefficient (0.3359), underscore the model’s effectiveness on this clinical dataset.

**Fig. 3:**
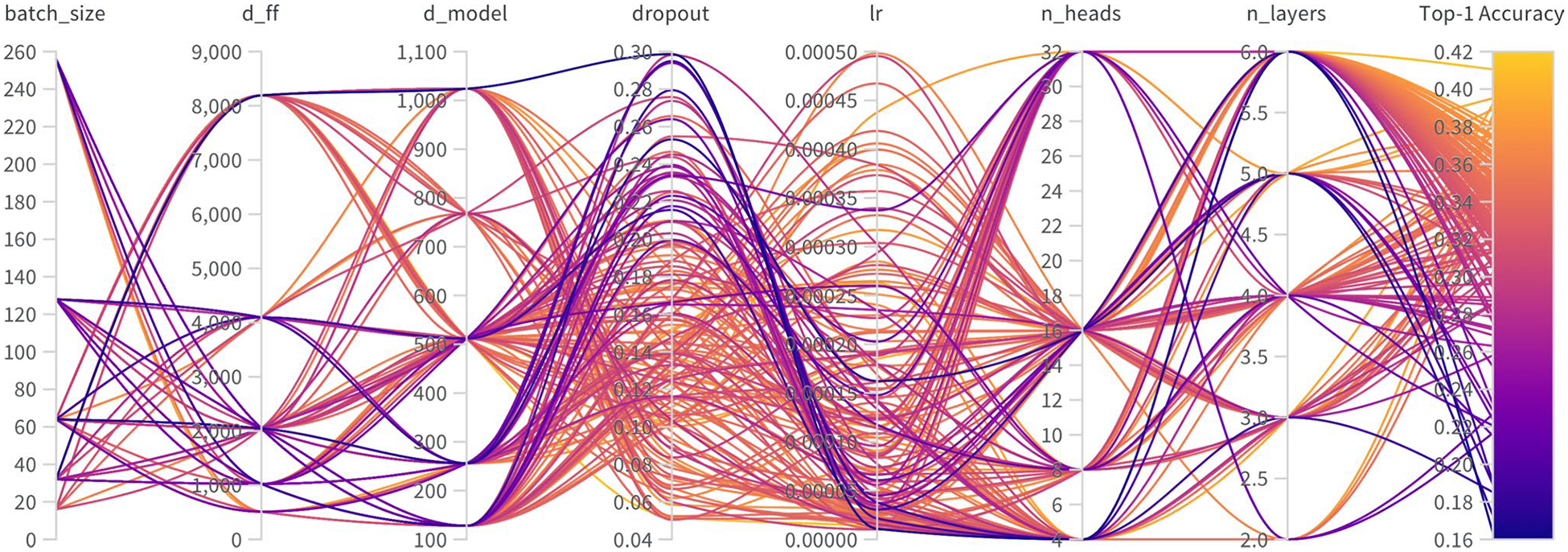
A parallel coordinates plot visualizing the hyperparameter optimization search. Each vertical axis represents a key hyperparameter, while each colored line traces a single experimental run. The line’s color corresponds to the final Top-1 Accuracy, with dark purple indicating the highest-performing models. This visualization reveals the configurations and parameter ranges that yield optimal model performance

Figure 3 shows the results of the hyperparameter optimization search, visualized as a parallel coordinates plot. Each line represents a single training run, mapping the values of seven key hyperparameters: batch_size, feed-forward dimension (d_ff), model dimension (d_model), dropout, learning rate (lr), number of attention heads (n_heads), and number of layers (n_layers). The color of each line corresponds to the resulting Top-1 Accuracy, as indicated by the color bar. This visualization illustrates the interplay between parameters and reveals that the highest-performing models (dark purple lines) are associated with specific parameter combinations, such as a higher number of layers and attention heads.

Upon fine-tuning and evaluating our model on the external MIMIC-IV dataset, it demonstrated not only strong generalizability but a marked improvement in performance across all metrics. The model achieved a Top-1 accuracy of 60.61%, with the Top-3 and Top-5 accuracies reaching 90.91% and 96.97%, respectively. This indicates a very high reliability in placing the correct diagnosis within a small set of predictions. The model’s confidence and ranking ability were similarly enhanced, evidenced by a low Perplexity of 6.16 and a high MRR of 0.767. Furthermore, its performance on the imbalanced dataset was exceptionally strong, achieving a Cohen’s Kappa of 0.537 and an MCC of 0.554.

Notably, these results are substantially higher than those from our initial test set. We attribute this significant performance increase to the nature of the MIMIC-IV dataset. As a large, well-structured database from a single hospital system’s, MIMIC-IV likely contains more standardized and less noisy disease progression pathways. This structured data environment appears to facilitate a more effective fine-tuning process, enabling the model to learn the underlying clinical patterns with greater precision.

#### 4.1.2 Training runtime and compute efficiency

The computational cost of model training was benchmarked on [Your GPU hardware, e.g., three NVIDIA V100 GPUs]. The runtime for a single training trial during the hyperparameter search varied, as expected, with model size. SPT-Small configurations were the fastest, with a median runtime of 658.0 s (mean 681.5 ± 277.6 s). SPT-Medium configurations had a similar median runtime of 655.0 s but showed higher variance (mean 778.4 ± 341.5 s). SPT-Large models, which ultimately performed best, were the most computationally intensive, requiring a median of 1146.5 s (approx. 19.1 min) per trial (mean 1076.5 ± 346.7 s).

#### 4.1.3 Effect of sequence length

With only a single prior diagnosis as context (predicting position 2), the model established a baseline performance with a Top-1 accuracy of 13.81% and a Top-3 accuracy of 30.64%. The model’s uncertainty was highest at this stage, as reflected by a Perplexity of 48.08. Performance improved markedly when the context grew to two prior diagnoses (predicting position 3). The Top-1 accuracy rose substantially to 24.77%, and Top-3 accuracy increased to 53.57%. This strengthening trend was corroborated by a sharp increase in MRR to 0.4138 and a significant drop in Perplexity to 19.63.

The model’s predictive power reached its peak when predicting the fourth token from a three-disease history. A dramatic performance gain was observed across all metrics, with Top-1 accuracy surging to its highest point of 46.06%. The model’s ability to place the correct diagnosis within a small set of possibilities also peaked, with Top-3 and Top-5 accuracies reaching 84.73% and 94.58%, respectively. All other metrics followed this trend, with MRR (0.6552), Kappa (0.3061), and MCC (0.3192) climbing to their maximum values. Concurrently, Perplexity fell to its lowest point of 6.33, signifying the highest level of model confidence.

**Fig. 4:**
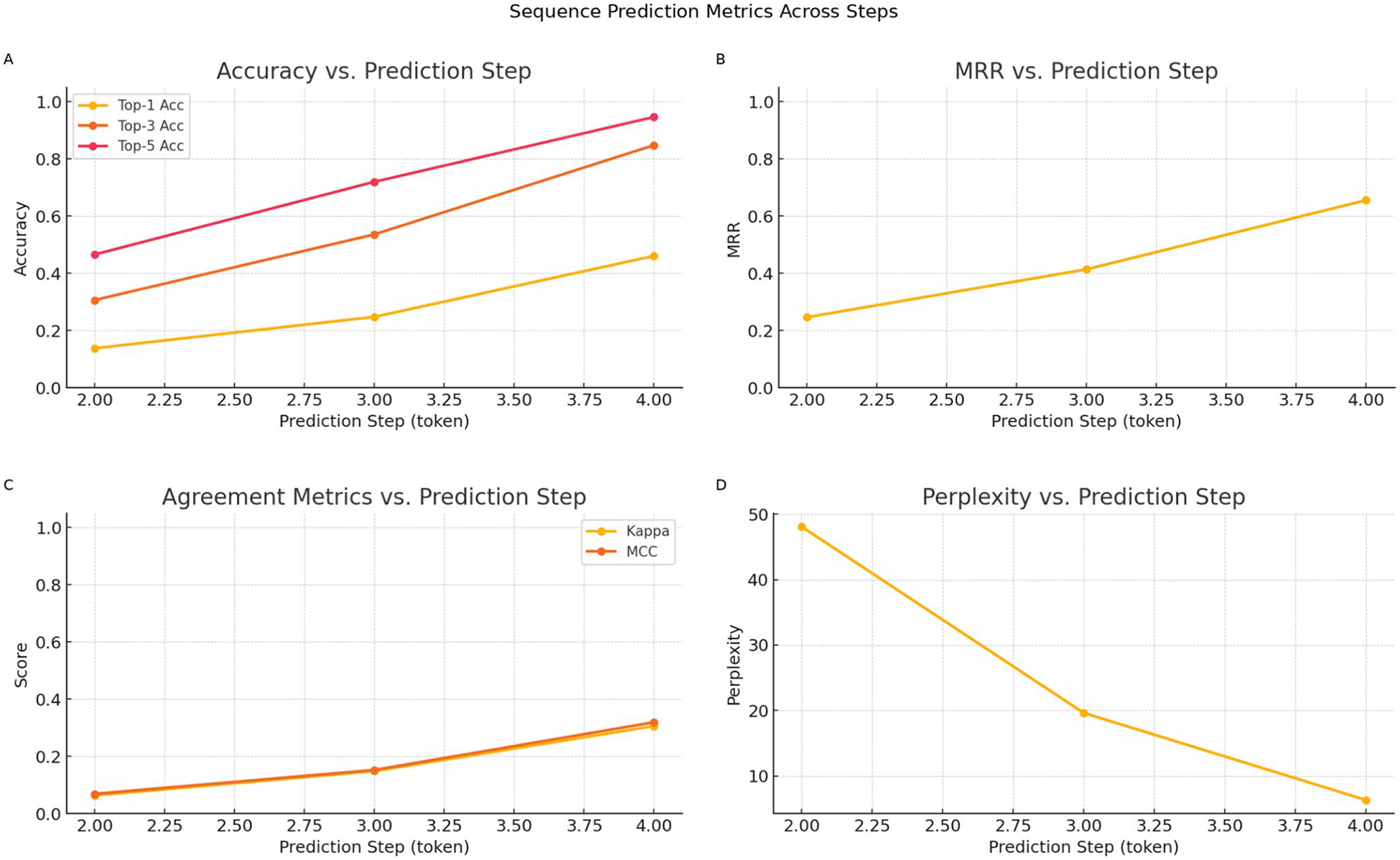
Impact of Historical Context on Prediction Metrics. The four panels illustrate the model’s performance as a function of the available sequence length when predicting the next diagnosis at steps 2, 3, and 4. **A** Top-1, Top-3, and Top-5 accuracy increase steadily as more context is provided. **B** Mean Reciprocal Rank (MRR) improves, indicating the model ranks the correct diagnosis higher with longer histories. **C** Agreement metrics (Kappa and MCC) rise, confirming robust performance. **D** Perplexity (PPL) decreases sharply, signifying greater model confidence. Collectively, the plots demonstrate a consistent and significant improvement in predictive power as the patient’s diagnostic history lengthens

These findings demonstrate a strong, positive correlation between the length of the diagnostic context and model performance. As more patient history becomes available, the model becomes progressively more accurate and confident in predicting the next step of a disease trajectory. This positive correlation between context length and model performance is visualized across all key metrics in Fig. 4.

**Fig. 5:**
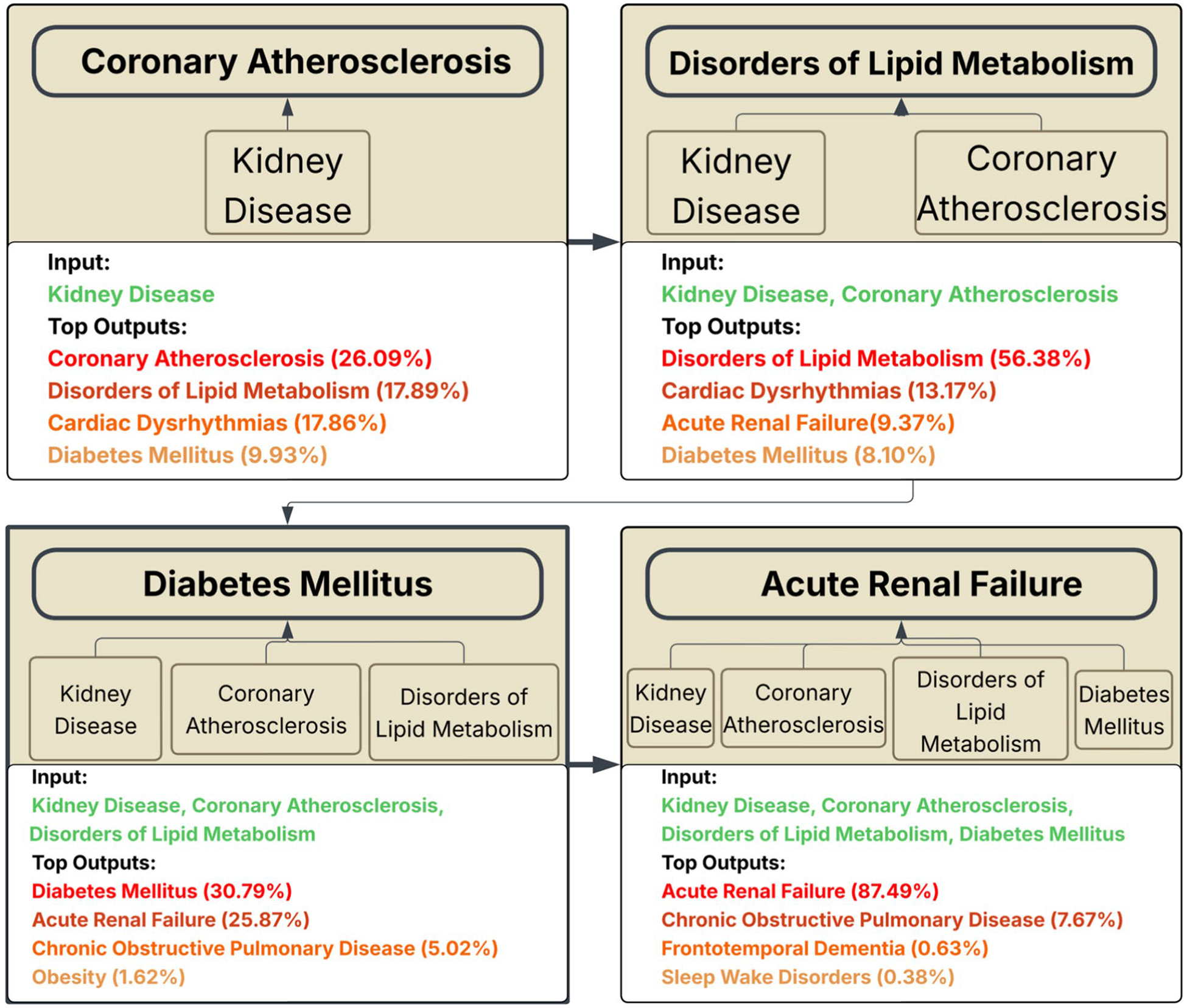
Case Study of a Sequentially Generated Disease Pathway. This figure illustrates the model’s dynamic reasoning across a four-step trajectory. Starting with an initial diagnosis of Coronary Atherosclerosis, each panel shows how the model’s top prediction evolves as the patient’s history accumulates, charting a clinically coherent path through cardiovascular and metabolic diseases that culminates in a high-certainty prediction of Acute Renal Failure

This complex behavior is best understood through the qualitative case study shown in Fig. 5, which traces a common and medically coherent disease trajectory, predicting specific, high-stakes clinical outcomes.

The model’s evolving predictions appear to mirror a physician’s diagnostic reasoning process. For instance, given an initial diagnosis of kidney disease, the model first predicts Coronary Atherosclerosis, reflecting the well-established clinical reality that chronic kidney disease is a major independent risk factor for cardiovascular events [42]. As the patient’s history grows to include atherosclerosis, the model logically infers an underlying Disorder of Lipid Metabolism as the next likely diagnosis, identifying the root cause of the atherosclerotic plaque buildup [43]. With a history of renal, cardiovascular, and metabolic dysfunction now established, the model astutely identifies Diabetes Mellitus, a systemic condition and cornerstone of cardiorenal metabolic syndrome that drives and exacerbates all prior pathologies [44]. Finally, presented with this full picture of chronic, multi-system illness, the model predicts Acute Renal Failure with overwhelming certainty (87.49%). This final prediction is clinically astute, as patients with long-standing diabetic and cardiovascular-related kidney damage are exceptionally vulnerable to episodes of acute-on-chronic renal failure [45]. This demonstrates that as the available clinical history lengthens, the model’s predictive capabilities mature. It progresses from identifying broad, high-frequency associations to capturing the more complex, higher-order conditional patterns that are indicative of specific clinical pathways.

### 4.2 Disease trajectory generation task

For the disease trajectory generation task, the standalone performance of the SPT model was evaluated on its ability to autoregressively generate sequences over a horizon of one, two, and three steps. The results, summarized in Table 4, reveal how the model’s performance evolves as it predicts further into a patient’s future.

As the generation horizon was extended from one to four steps, the model’s Perplexity progressively increased from 18.87 to 61.07. This trend is expected and indicates that the model’s certainty in its predictions decreases as it is tasked with forecasting further into an unknown trajectory. A corresponding decline was observed in factual accuracy. The Sequence Overlap score, measuring the proportion of correctly predicted diseases, dropped from 38.4% at the first step to 20.8% by the fourth step. This highlights the inherent and increasing difficulty of maintaining high precision over longer generative sequences. In contrast, metrics evaluating the structural coherence of the generated pathways remained largely stable. The BLEU-1 score began at 38.43% and ended at 37.33%, and the ROUGE-L score showed a similar pattern. This suggests that while the model’s confidence and ability to predict the specific correct diseases diminish over a longer horizon, its capacity to generate structurally sound and plausible disease pathways is well-maintained. The model successfully learns the underlying “grammar” of disease progression, even as the task’s complexity increases with each step.

While these quantitative metrics establish the model’s predictive performance, a deeper analysis is required to understand the clinical plausibility of its generated trajectories. Our qualitative analysis confirms that the SPT model successfully learns clinically relevant patterns, often generating pathways that adhere to known principles of disease progression. A full exploration of the model’s interpretability, complete with case studies and visualizations that reveal its internal logic, is presented in the forthcoming Discussion section.

## 5 Discussion

In this study, we developed the SPT, a novel framework that integrates sequential pattern mining with a transformer architecture to model the complex progression of type 2 diabetes. Our results demonstrate the success of this method on two levels. First, in terms of overall predictive performance, the SPT model achieved an average Top-1 accuracy of 41.23% and a Top-5 accuracy of 85.78%, outperforming strong recurrent neural network baselines, including LSTM (31.65% Top-1 accuracy). Second, and more importantly, our findings show that the model successfully emulates a nuanced clinical reasoning process when analyzing patient histories of varying complexity. A key finding was the evolution of the model’s performance with increasing sequence length. The model’s ability to leverage additional disease context and patient history reflects a critical clinical reality: as a patient’s condition becomes more chronic and multi-faceted, identifying a narrow set of high-risk possibilities becomes more valuable than predicting a single definitive event.

A key innovation of our SPT framework is the foundational use of sequential pattern mining prior to model training. This step serves as a critical data regularization and de-noising mechanism that offers a dual benefit. First, by training the model on frequent, statistically validated disease pathways rather than the full, noisy spectrum of all patient histories, it makes the computationally demanding process of training a transformer more efficient and tractable. Second, this approach grounds the model in established clinical reality, mitigating the risk of overfitting to spurious correlations. In essence, pattern mining acts as a domain-specific inductive bias, guiding the transformer to focus on the most meaningful patterns, which enhances model robustness and contributes to its ability to generate clinically coherent predictions.

The transformer’s self-attention mechanism enables the model to consider the entire patient history, weighing the relevance of distant events to understand the patient’s overall state. Combined with our decoder-only (GPT-style) architecture, the SPT framework enables both accurate next-disease prediction and the simulation of clinically coherent trajectories, addressing the broader needs of clinical decision support beyond simple risk scores. To better understand the basis for these results, we now turn to a detailed interpretation of the model’s learned behaviors.

### 5.1 Interpretation

A central achievement of this study is the SPT model’s sophisticated ability to understand and process disease sequences in a manner that mirrors clinical reality. This interpretation can be deconstructed into three key layers: the model’s foundational knowledge of disease relationships, its dynamic capacity to map and compare different health trajectories, and the transparency of its decision-making process for any single prediction.

#### 5.1.1 Disease relation

**Fig. 6:**
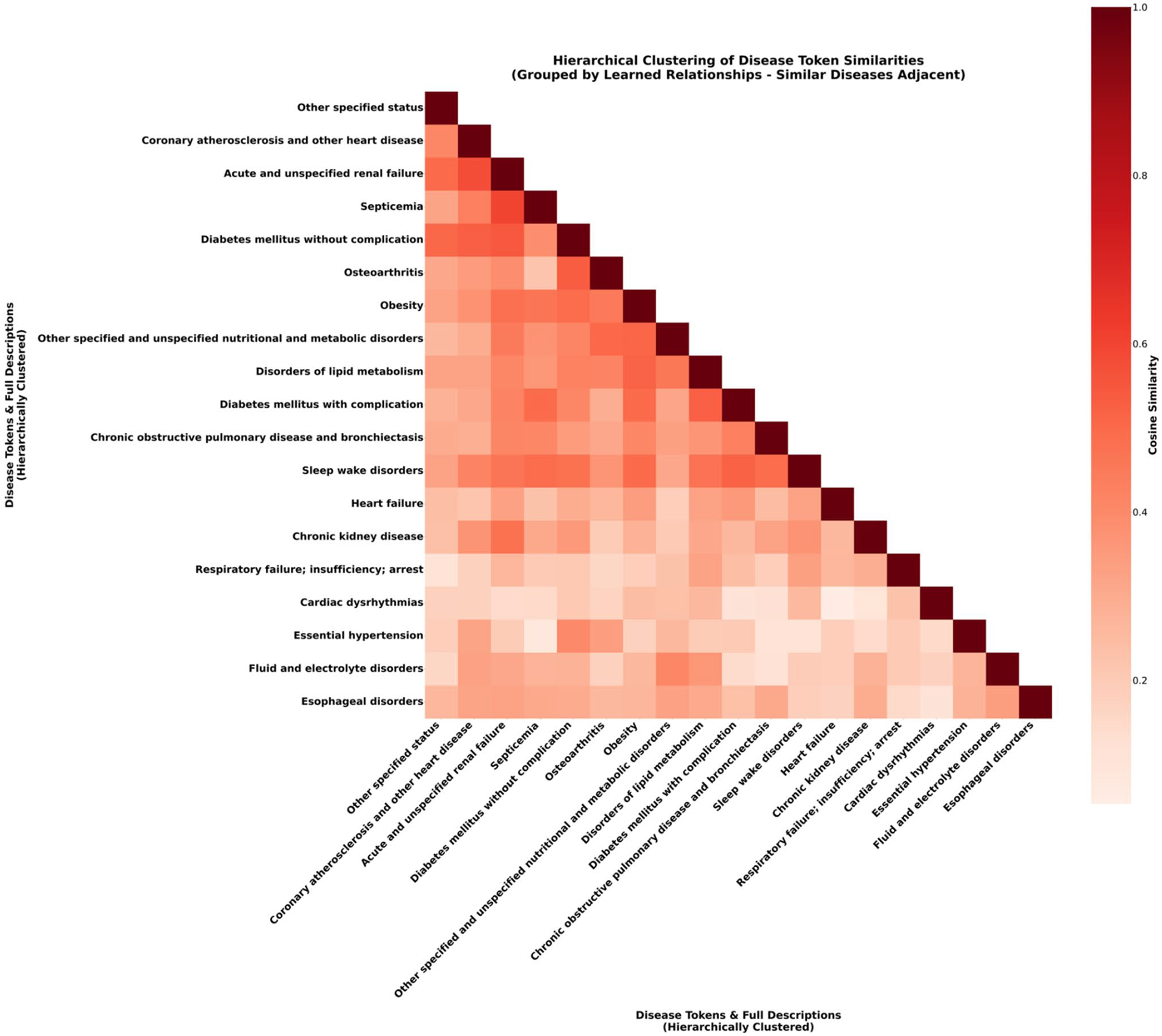
Heatmap of Learned Disease Token Similarity After Hierarchical Clustering. Disease token similarity matrix based on last layer representations with full CCSR descriptions, showing cosine similarity values between different disease categories. The matrix displays varying degrees of similarity (ranging from 0 to 1.0 as indicated by the color scale) among 18 disease tokens including disorders of lipid metabolism, diabetes mellitus with complications, chronic kidney disease, coronary atherosclerosis, heart failure, and other cardiovascular, metabolic, and systemic conditions. The axes are reordered by a hierarchical clustering algorithm, ensuring that semantically similar diseases appear adjacent to one another. Darker red regions indicate higher similarity between disease tokens, while lighter yellow regions represent lower similarity, revealing learned relationships between different medical conditions in the model’s embedding space

To validate the model’s foundational knowledge, we visualized the semantic relationships it learned between diseases (Fig. 6 Heatmap). This was achieved by extracting the final-layer vector representations, or “embeddings,” that the SPT model learned for each unique disease token. We then calculated the cosine similarity between every pair of these disease vectors to create a comprehensive similarity matrix. We then applied hierarchical clustering to reorder this matrix, grouping similar diseases together to reveal clinically meaningful relationships in the final heatmap visualization. Clustering method ensures that diseases the model considers similar are placed adjacent to each other, making clinically related groups visually apparent in the final heatmap. This analysis provides compelling evidence that the model has independently learned to organize diseases according to their clinical and pathophysiological connections. The most prominent feature is the strong cluster of high similarity linking Diabetes Mellitus, Chronic Kidney Disease, Disorders of Lipid Metabolism, and Coronary Atherosclerosis which is well documented [46]. This visually confirms the model discovered the core components of cardiorenal metabolic syndrome, while its ability to distinguish these from unrelated conditions like Osteoarthritis demonstrates a clinically valid and specific understanding.

#### 5.1.2 Different pathways in healthcare

**Fig. 7:**
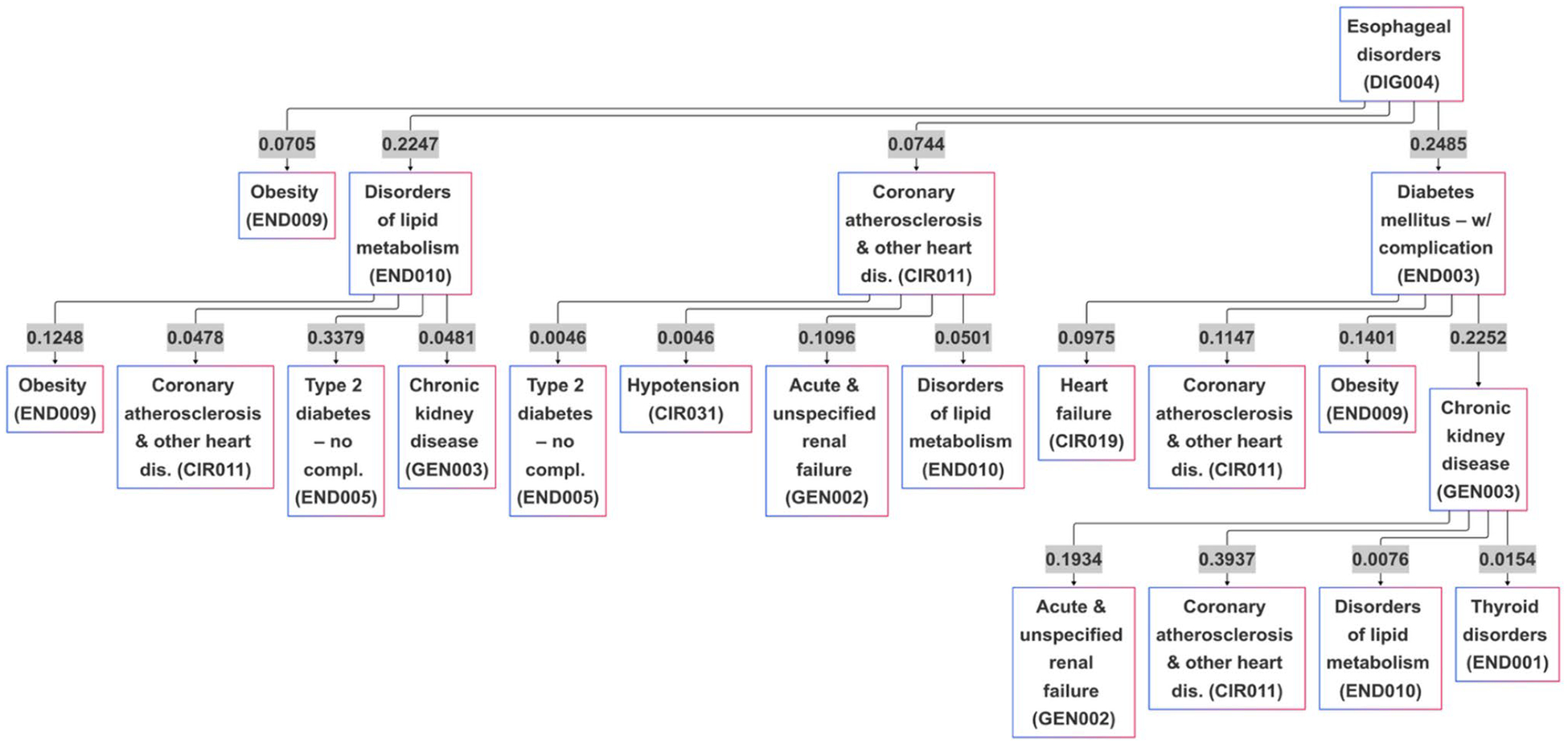
A data-driven “atlas” of disease progression generated by the SPT model, visualizing the most common probabilistic pathways originating from an initial diagnosis of Esophageal disorders and branching into subsequent metabolic, renal, and cardiovascular complications. The flowchart displays conditional probabilities at each branch point, showing how patients may progress from esophageal disorders (DIG004) to obesity (END009) with probability 0.2488, then through various pathways including coronary atherosclerosis and heart disease (CIR011), diabetes complications (END005), chronic kidney disease (GEN003), and other cardiovascular conditions. Each arrow is labeled with transition probabilities, illustrating the likelihood of developing specific comorbidities based on the learned disease progression patterns from patient data

Building on this coherent knowledge base, the model can generate a comprehensive, probabilistic “atlas” of potential disease trajectories (Fig. 7). For example, this map reveals that from a starting point of esophageal disorder, a condition increasingly recognized as a gastro-metabolic nexus in type 2 diabetes cohorts, the dominant progression is towards diabetes with complications, occurring with a 24.9% probability. This structure aligns with epidemiological evidence that gastroesophageal disease frequently co-occurs with diabetes [46]. From this critical inflection point, the model charts a clinically validated cascade: the most likely next event is chronic kidney disease (22.5% probability), followed in turn by a 39.4% transition risk to coronary atherosclerosis, and then a 19.3% risk of acute renal failure. These figures mirror contemporary cohort data showing that diabetic-chronic kidney disease (CKD) overlap amplifies cardiovascular and renal events [47, 48] and that optimal, guideline-directed cardio-renal therapy at this juncture improves both heart and kidney outcomes [49].

This data-driven pathway transforms a static diagnosis into an actionable, time-ordered map. By spotlighting high-probability transitions, such as the progression from diabetic kidney disease to cardiovascular events, it provides clinicians with clear windows for targeted interventions, for instance, the early initiation of therapies like SGLT2 inhibitors or GLP-1RAs, which are now known to provide disease-modifying benefits at exactly these stages [50]. Ultimately, this generative map functions as a data-driven “atlas” of disease, operationalizing AI-enabled temporal phenotyping to move clinical decision-making from reactive event management to proactive, trajectory-based precision care.

**Fig. 8:**
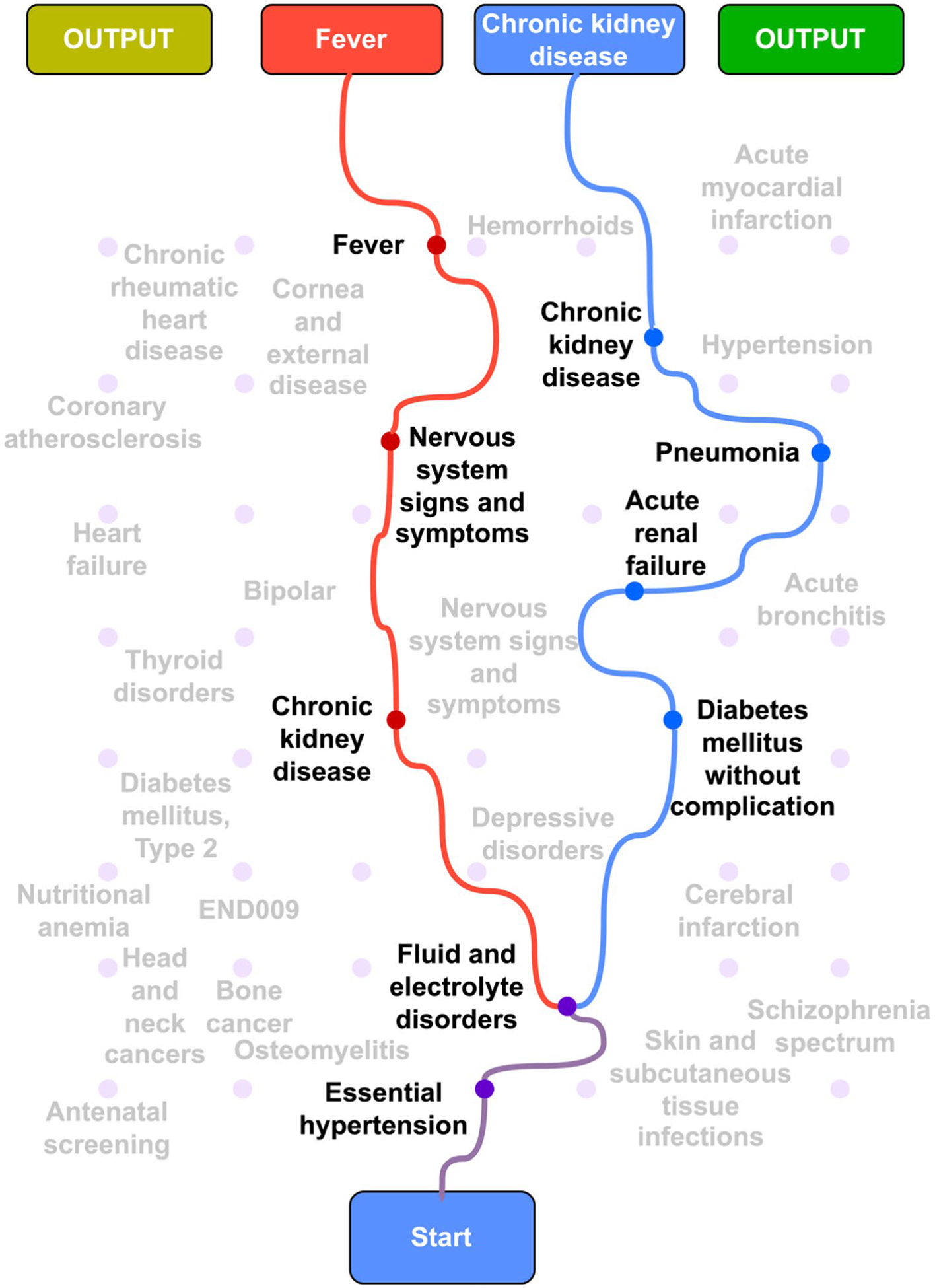
Counterfactual Simulation of Diverging Disease Pathways. This graph simulates how a single differing diagnosis creates two distinct patient futures. Both trajectories share a common start (Essential hypertension → Fluid and electrolyte disorders) before splitting. The blue path shows a metabolic-renal cascade initiated by Diabetes, leading to Chronic kidney disease. In contrast, the red path follows an inflammatory trajectory culminating in Fever, demonstrating the model’s ability to identify critical, path-defining clinical events

Beyond mapping common pathways, the model’s true power for personalized medicine is revealed in its ability to simulate how these trajectories diverge based on single clinical events (Fig. 8). Figure 8 demonstrates the path-dependent nature of disease evolution in clinical practice, wherein all patients in the cohort initiate through a common diagnostic pathway of essential hypertension progressing to fluid-and-electrolyte disorders. However, at this critical juncture, the emergence of a single alternate diagnosis fundamentally alters the subsequent disease trajectory.

When the next diagnostic label is diabetes mellitus without complication, the clinical sequence follows a metabolic-renal pathway, characterized by rapid progression through acute renal failure and pneumonia before culminating in CKD. This progression is supported by large registry studies demonstrating that type 2 diabetes nearly doubles the risk of acute kidney injury, particularly when complicated by community-acquired pneumonia [51], with both conditions serving as potent catalysts for incident CKD [52]. This trajectory represents a clinically actionable decision point where early initiation of SGLT-2 inhibition and implementation of stringent infection prophylaxis measures may alter disease progression [50].

Alternatively, when the diagnostic pathway diverges from fluid and electrolyte disorders toward CKD, the model predicts a fundamentally altered disease trajectory. This trajectory identified by the model follows a sepsis-neuro-renal cascade that is well-substantiated by clinical literature. The sequence begins with Essential Hypertension, where first-line diuretic therapy is a leading cause of the subsequent diagnosis, Fluid and Electrolyte Disorders [53, 54]. The model then links this electrolyte disturbance to the onset of Fever, which aligns with evidence that such imbalances are often early markers for underlying sepsis [55]. Subsequently, the model connects established CKD to Nervous-System Signs and Symptoms, consistent with the spectrum of neuro-cognitive complications driven by uremic toxins [56]. The final step to Recurrent Fever highlights the compromised immune state of patients with CKD, which elevates their risk of subsequent infection [57]. This step-by-step validation demonstrates that the model identifies not just statistical correlations, but a pathophysiological inter-locking chain of events, confirming its ability to recognize and project complex, real-world clinical narratives.

The clinical implication of being able to generate and compare these pathways is profound. It transforms the model from a passive forecasting tool into an interactive decision support system. Clinicians can explore “what-if” scenarios, effectively asking the model to project the consequences of a new diagnosis or the potential benefits of a targeted intervention. This allows for a highly personalized approach to preventive medicine, where strategies can be tailored based on a patient’s unique trajectory. By quantifying how a single change can reroute a patient’s entire future, our model provides a powerful, data-driven method for visualizing risk and optimizing clinical decision-making over the long course of chronic illness management.

#### 5.1.3 Explainable AI for clinical decision support

The final layer of interpretation addresses the “black box” problem. To ensure the model’s predictions are transparent and clinically trustworthy, we employed SHapley Additive exPlanations (SHAP), a state-of-the-art XAI method derived from cooperative game theory [58]. For any given prediction, SHAP calculates the marginal contribution of each input feature, in our case, each past disease in a patient’s trajectory. It assigns a “SHAP value” to each past disease, quantifying precisely how much that specific condition pushed the final prediction score higher (a positive SHAP value) or lower (a negative SHAP value). This technique allows us to deconstruct the “black box” nature of the model and provide a clear, evidence-based justification for any individual forecast.

**Fig. 9:**
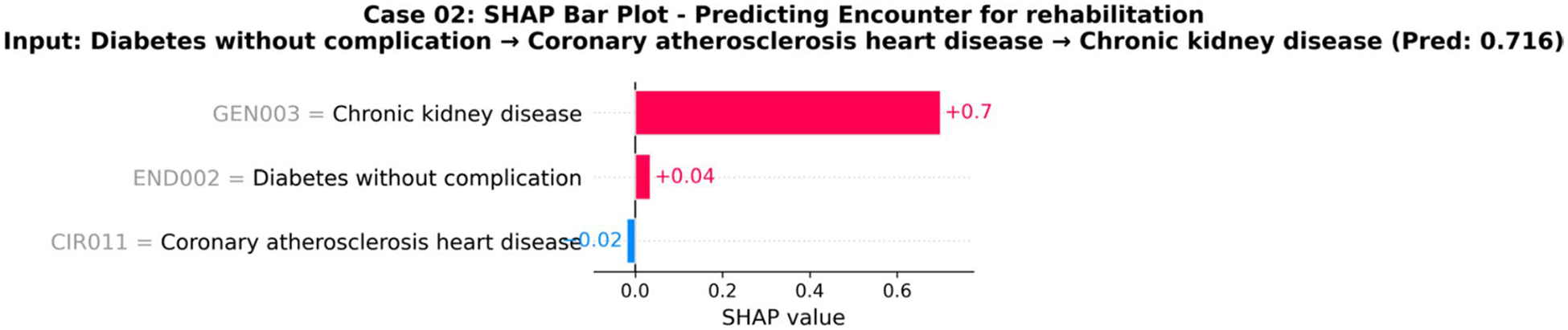
Explainable AI (XAI) Analysis of an Individual Prediction using SHAP. SHAP bar plot for Case 02 showing feature contributions to predicting an encounter for rehabilitation, given an input sequence of diabetes without complication → coronary atherosclerosis heart disease → chronic kidney disease (prediction confidence: 0.716). The plot reveals that chronic kidney disease (GEN003) has the strongest positive contribution (+ 0.7 SHAP value) toward the prediction, diabetes without complication (END002) provides a modest positive contribution (+ 0.04), while coronary atherosclerosis heart disease (CIR011) has a slight negative contribution (− 0.02). The analysis demonstrates how different disease tokens in the patient’s history influence the model’s prediction for the next medical encounter

Figure 9 shows an XAI analysis of single prediction using SHAP. SHAP analysis identifies chronic kidney disease as the primary driver of general rehabilitation referrals, with a substantial positive contribution of + 0.70. CKD’s associated burden of anemia, sarcopenia, and exercise intolerance necessitates multidisciplinary rehabilitation including strength training, intradialytic exercise, and nutrition counseling as the evidence-based standard of care [59, 60]. Diabetes mellitus contributes a modest positive influence (+ 0.04) through impaired muscle quality and wound healing associated with poor glycemic control. Conversely, coronary atherosclerosis demonstrates a slight negative effect (− 0.02), as these patients are typically directed toward cardiac-specific rehabilitation programs rather than general rehabilitation services [61]. The SHAP values thus reflect established clinical practice patterns, with CKD as the predominant indication for general rehabilitation. This suggests the model has learned to differentiate primary drivers from associated comorbidities, correctly identifying the renal condition, not the cardiac history, as the most direct reason for a general rehabilitation need in this patient’s context. By providing this level of transparent, clinically sound reasoning, the model builds trust and can serve as a true collaborative tool for physicians.

Our research significantly advances the field of diabetes management, both theoretically and practically. By integrating sequential pattern mining with transformer-based models, we achieve more accurate and nuanced predictions of diabetes complications compared to existing methods. Moreover, the enhanced interpretability offered by SHAP visualizations addresses a critical need in clinical AI applications, paving the way for responsible and effective AI integration in healthcare. While these advancements represent important contributions to the field, they also highlight opportunities for further innovation and refinement.

### 5.2 Implications

The findings of this study carry specific implications that are directly rooted in the novel capabilities of the SPT model. The most significant implication arises from the model’s dynamic response to increasing patient complexity. Our result that the model shifts from high Top-1 accuracy in earlier disease stages to high Top-5 accuracy in later, more complex cases implies that a single AI tool can serve two distinct clinical functions. For patients with fewer comorbidities, it can act as an early warning system for the single most likely complication. For patients with advanced multi-morbidity, its utility evolves into a powerful tool for generating a differential diagnosis, helping clinicians navigate ambiguity by focusing on a small, accurate set of high-risk possibilities. This adaptability to case complexity represents a major advance over static clinical risk scores.

Furthermore, the model’s generative capabilities, as shown in the multi-pathway trajectory tree, have profound implications for strategic health management that extend beyond individual patients. The ability to generate a probabilistic “atlas” of potential futures implies that health systems could use this technology to map the most common “highways” to severe complications within their entire population. This allows for data-driven public health strategies and system-level interventions focused on high-yield prevention points. For the individual clinician, the ability to simulate counterfactuals, visualizing how an intervention might alter a patient’s future, implies a shift toward a more interactive and evidence-based dialogue, strengthening both the physician’s confidence in a care plan and the patient’s adherence to it.

Additionally, the transparent reasoning demonstrated by our model has direct implications for clinical adoption and trust. The combination of a clinically coherent semantic space (verified by the disease similarity heatmap) and patient-specific prediction explanations (provided by SHAP analysis) ensures that the model’s outputs are interpretable. This implies a new paradigm for human-AI collaboration, where a clinician can trust a prediction because they can verify its underlying logic, both at a macro level (seeing that the model understands cardiorenal metabolic syndrome) and at a micro, patient-specific level. This verifiable reasoning is the critical catalyst needed to translate predictive models from research papers into indispensable tools at the clinical front line.

A key implication of this work is the generalizability and adaptability of the SPT framework. Our “pattern-guided” methodology is domain-agnostic, which implies that the SPT framework could serve as a transferable and interpretable modeling strategy for a broad spectrum of clinical conditions, not just chronic diseases. This approach is applicable to any domain characterized by sequential progression, ranging from long-term chronic management (e.g., COPD, heart failure) to acute care pathways or infectious disease modeling, provided sufficient longitudinal data is available. Furthermore, the model is highly adaptable within a specific disease domain. A pre-trained SPT model, like the one developed here, could be efficiently fine-tuned on smaller, local datasets from different hospital systems. This fine-tuning would allow the model to adapt to a new institution’s specific patient demographics and clinical coding practices, significantly enhancing its local predictive accuracy and clinical utility. This makes the SPT framework a transferable, adaptable, and robust approach for modeling diverse disease trajectories across complex medical settings.

### 5.3 Limitations

This study’s primary limitation is its dataset, a four-year window of inpatient-only records (SID), which provides deep insight into acute exacerbations but lacks the long-term outpatient history of chronic illness. Consequently, our model is highly effective for predicting pathways toward severe, hospital-worthy events. Its applicability to modeling the slower, decade-spanning trajectories managed in outpatient settings remains a key opportunity for future research and defines the scope of our current findings.

In addition, our methodology concentrates on common disease trajectories, identified via the PrefixSpan algorithm, to ensure the model is robust and effective for the pathways affecting the majority of patients. This deliberate design choice means the model is not intended for rare diseases or highly atypical patient journeys. Modeling such cases is a distinct and important challenge that requires specialized datasets and methods, representing a separate, critical avenue for future research.

### 5.4 Future works

Building directly upon the findings and limitations of this study, future work should focus on creating a more holistic model of disease progression. This will provide a complete, multi-year view of the patient journey, enabling the model to learn the slower, long-term dynamics of chronic illness. With this comprehensive data, the SPT framework can then be enhanced with time-aware attention mechanisms to explicitly model the intervals between clinical events. Ultimately, the framework’s real-world value must be assessed through prospective clinical trials to determine if its predictive power translates into measurable improvements in patient outcomes.

## 6 Conclusions

In this study, we addressed the critical challenge of reactive chronic disease management by developing the SPT, a framework that synergistically combines the strengths of sequential pattern mining and a generative transformer architecture. Our work demonstrated that this integrated approach produces more than just an accurate predictor; it yields a dynamic reasoning system capable of adapting its function from single-event forecasting in early disease to generating a precise differential diagnosis as patient complexity increases. Crucially, we showed that the model’s predictions are not opaque, but are grounded in a learned, clinically coherent knowledge base and are explainable at the individual patient level. The SPT framework therefore represents a significant step toward overcoming the critical barriers of trust and utility in clinical AI. It provides a validated blueprint for a new generation of tools designed not just to forecast risk, but to provide transparent, generative, and actionable foresight, empowering clinicians to finally transition to a proactive and personalized standard of care.

## Figures and Tables

**Table 1:** Patient demographics for all the patients with E11.X disease codes

Patient with diabetes		
Number of patients	258,460	
Age mean [0.25,0.75]	66.15 [57.0, 77.0]	
Gender	#	%
Male	128,185	49.60%
Female	130,274	50.40%
Race		
White	136,691	52.89%
Black	96,782	37.45%
Hispanic	10,028	3.88%
Asian or Pacific Islander	6819	2.64%
Native American	771	0.30%
Other	5346	2.07%
Ethnicity		
Not hispanic	239,394	92.62%
Hispanic white	2765	1.07%
Hispanic black	495	0.19%
Hispanic other	6574	2.54%
Hispanic unspecified	194	0.08%
Insurance		
Medicare	155,572	60.19%
Medicate	32,429	12.55%
Private	60,537	23.42%
Self-pay	3267	1.26%
No charge	312	0.12%

**Table 2:** Hyperparameter search spaces

Hyperparameter	Small models	Medium models	Large models
Embedding	[128, 256]	[256, 512]	[512, 768, 1024]
Transformer layers	2,4	4,6	6, 8, Or 16
Feed-forward	[512, 1024, 2048]	[1024, 2048, 4096]	[2048, 4096, 8192]
Attention heads	[4, 8, 16, 32]	[4, 8, 16, 32]	[4, 8, 16, 32]
Batch size	[128, 256, 512]	[64, 128, 256]	[32, 64, 128]
Dropout rate	[0.10, 0.30]	[0.10, 0.30]	[0.05, 0.30]
Learning rate (Lr)	[3e–5, 5e–4]	[2e–5, 3e–4]	[1e–5, 2e–4]

**Table 3:** Performance comparison for next disease prediction task

Model	Top-1 accuracy (%)	Top-3 accuracy (%)	Top-5 accuracy (%)	MRR	PPL	Kappa	MCC
RNN [39]	26.36	50.90	63.57	0.4406	24.60	0.1174	0.1291
GRU[40]	17.77	44.16	57.11	0.3724	23.16	0.0313	0.0487
LSTM [41]	31.65	56.53	71.47	0.4850	28.71	0.1951	0.2092
SPT_LARGE_	**41.23**	**72.51**	**85.78**	**0.5897**	**12.34**	**0.3027**	**0.3359**
SPT_MEDIUM_	41.06	66.66	79.71	0.5729	13.24	0.2504	0.3245
SPT_SMALL_	39.90	65.11	79.07	0.5682	13.10	0.2606	0.3133

The bold value in table indicate the best highest performing

**Table 4:** Performance comparison for disease trajectory generation task

Horizon	Perplexity	Seq Overlap	BLEU-1	ROUGE-L
1	18.87	38.4	38.43	38.43
2	27.85	27.8	38.08	38.08
3	43.10	21.3	35.23	35.21
4	61.07	20.8	37.33	37.27

## Data Availability

The data analyzed in this study are derived from the State Inpatient Database (SID), which is part of the Healthcare Cost and Utilization Project (HCUP), sponsored by the Agency for Healthcare Research and Quality (AHRQ). Access to this data is governed by a Data Use Agreement (DUA), which prohibits the authors from redistributing the dataset. Further information on accessing SID data can be found at: https://hcup-us.ahrq.gov/sidoverview.jsp. The source code is available at: https://github.com/Luo-Innovation-Lab/SPT.
